# Distress and Spiritual Well-Being in Brazilian Patients Initiating Chemotherapy during the COVID-19 Pandemic—A Cross-Sectional Study

**DOI:** 10.3390/ijerph182413200

**Published:** 2021-12-15

**Authors:** Angelo Braga Mendonça, Eliane Ramos Pereira, Carinne Magnago, Pedro Gilson da Silva, Diva Cristina Morett Leão, Rose Mary Costa Rosa Andrade Silva, Karina Cardoso Meira

**Affiliations:** 1 Nursing School, Fluminense Federal University, Niterói 24020-091, Brazil; elianeramos.uff@gmail.com (E.R.P.); divaleao@yahoo.com.br (D.C.M.L.); roserosa.uff@gmail.com (R.M.C.R.A.S.); 2 School of Public Health, University of São Paulo, São Paulo 01246-904, Brazil; carinne@usp.br; 3 School of Health, Federal University of Rio Grande do Norte, Natal 59075-000, Brazil; csc.pns.2019@gmail.com (P.G.d.S.); ninameira87@gmail.com (K.C.M.)

**Keywords:** cancer, chemotherapy, psychological distress, spirituality, COVID-19

## Abstract

Social distancing and the priority given to COVID-19 patients in health services, which caused postponement of appointments and cancer treatment, may have triggered unprecedented levels of distress in cancer patients. The aim of this study was to analyze the prevalence of distress and the levels of spiritual well-being of people initiating chemotherapy during the COVID-19 pandemic, identifying the factors associated with distress, and determining if there is a relationship between distress and spiritual well-being. A cross-sectional study was conducted with 91 Brazilians. Data were collected by applying the Spiritual Well-Being Scale (SWBS) and the Distress Thermometer and Problem List for Patients. The prevalence of distress was 59.5%, and the average score of spiritual well-being was 106.54 (±9.06). Emotional issues were the most reported by patients with distress. The Poisson regression showed that male sex (PR = 0.588; 95% CI 0.392–0.881), age (PR = 0.985; 95% CI 0.973–0.996), and spiritual well-being score were predictors of distress (PR = 0.971; 95% CI 0.946–0.996). These findings indicate that distress relief involves implementation of public health programs capable of integrating spiritual interventions into cancer care.

## 1. Introduction

Recent studies have pointed out different negative impacts of coronavirus disease 2019 (COVID-19), such as a marked increase in levels of psychological distress in the population in general [1] and increases in the mortality of patients with cancer [2]. However, evidence is lacking about the complex factors in perception of distress and spiritual well-being in patients who started chemotherapy during the pandemic period. Despite the urgency of treatment for extending patient survival, many chemotherapy procedures have been delayed in an attempt to manage COVID-19 cases, thus creating a dilemma for cancer care in epicenters of the disease, including Brazil, where more than 600,000 people have died of SARS-CoV2 [3,4]. 

In the battle against cancer, over 2,644,897 chemotherapy treatments are carried out every year in Brazil [5], and they account for more than 48% of the costs of cancer therapy in the Brazilian Unified Health System. Chemotherapy has increasingly been administered as both outpatient and inpatient treatment. It differs from radiotherapy and surgery because it is a systemic, intense, and cyclic treatment modality whose completion takes a long time and requires frequent hospitalizations. Despite its importance in healing some types of neoplasia, controlling symptoms, and increasing survival, its adverse effects can affect patients’ psychological and spiritual states [6,7,8,9].

The feeling of lack of control over the situation can be stronger before the first chemotherapy cycle, originating in erroneous conceptions disseminated in the media and negative experiences of relatives and friends. As a consequence, a distorted picture can be created because of overestimation of adverse effects, which results in increased mental stress, fatigue, sleeplessness, pain, and increased risk of anticipatory nausea and vomiting [10,11,12].

Given these psychological consequences, for the cancer context, distress was defined as a feeling of threat that varies over a continuum. Beginning with normal feelings of vulnerability, sadness, and fear, it can reach alarming proportions when depression, anxiety, demoralization, and social distancing lead to disability and suicidal ideation. This experience is multidimensional and pervaded by social, physical, and spiritual aspects that interfere with the ability to positively deal with the diagnosis and the necessary therapies [13,14,15]. Often confused with the concept of stress, the term “distress” represents a perception of inability to deal with challenging situations. This results in profound changes in the emotional state of patients and can cause maladaptive responses. For distress to occur, a stressor or unmet demand is necessary. From this perspective, stress can be understood as an antecedent of distress, being defined as a response that is not necessarily negative in nature. Therefore, stress encompasses, on the psychological and physiological levels, any situation of unpredictability and uncontrollability that exceeds the person’s regulatory capacity. From this point of view, stress is an important driver of life, while distress, if not treated, can negatively affect patients’ overall survival and quality of life, and the results of chemotherapy [16,17,18].

The epidemiological data have shown that more than half of cancer patients may develop moderate to intense psychological stress in some phase of the disease [19]. Many studies have focused on evaluating this phenomenon by using different measuring instruments in an attempt to identify common practical problems [7,17,20]. Some authors have shown that distress can be influenced by sociodemographic and clinical variables [21,22,23,24]. Others have found no association between these variables [25,26]. These differences may be the result of factors that are not entirely understood, such as willingness to report distress, cultural aspects, observation bias, and the subjective nature of the concept [10,22,27,28,29]. 

In addition, several factors that have not been measured in previous studies, including religiosity and spirituality, may shape the relationship between distress and different variables. Differentiation of these concepts proved to be useful to show how individuals use resources from the religious and spiritual dimensions to control levels of distress. Spirituality provides a means for individuals to find meaning in distress through connections to the sacred, self, and life. In the religious dimension, they access their faith, practice rituals, and use the support of the church and their relationship with God to adapt to the new situation. Mixed, and even controversial, results about the effects of religiosity and spirituality in this process show that it is still unclear how these dimensions fit into the way patients react to situations that challenge their spiritual beliefs [10,30,31,32]. 

In these times of uncertainty, concerns such as fear of cancer progression and physical disabilities may have been intensified by the expectation of developing COVID-19 and dying. Contingency plans were designed, in both the study location and other institutions, to stop the advance of the pandemic. However, it is believed that safety measures, such as postponing surgeries and chemotherapy treatments, blocking the use of hospital beds for patients other than COVID-19 patients, carrying out online appointments, and preferring prescription of oral antineoplastic drugs, have triggered unprecedented levels of distress in patients in the first phases of their chemotherapy treatment [3]. Furthermore, studies have indicated that control measures such as stay-at-home requests, social distancing, and temporary closing of religious institutions have negatively impacted patient well-being, especially its spiritual aspects [33,34], which may have triggered or aggravated the distress.

Considering the effects of the COVID-19 pandemic on mental and spiritual well-being, the increased vulnerability of patients who are initiating chemotherapy, and the multiplicity of variables that affect the phenomenon under discussion, the authors of the present study decided to explore its most important correlatives. In places where time and human resources are limited, screening patients who show higher risks of developing distress becomes important [35]. Knowing these factors can help health teams identify more vulnerable groups, which the literature has identified as more likely to withdraw from treatment, miss chemotherapy sessions, and evolve into extreme consequences, such as suicide [7,24,36]. Consequently, this information can lead to progress in the development of strategies to prevent and relieve psychological distress.

In view of the above, the authors carried out a study with patients who were initiating chemotherapy in Brazil, a country that has been an epicenter of the COVID-19 pandemic. Its objectives were analyzing the prevalence of distress and the levels of spiritual well-being of people initiating chemotherapy during the COVID-19 pandemic, identifying the factors associated with distress, and determining if there is a relationship between distress and spiritual well-being.

## 2. Materials and Methods

### 2.1. Study Design, Population, and Sample

This was a cross-sectional study carried out from December 2020 to March 2021. The Strengthening the Reporting of Observational Studies in Epidemiology (STROBE) Statement was applied (see Supplementary Materials).

The population was adult patients who had a confirmed diagnosis of neoplasia and were initiating chemotherapy. The study location was the outpatient clinic of the Brazilian National Cancer Institute (INCA), a national reference hospital for cancer education and control, located in the city of Rio de Janeiro, Brazil. The offered services are free, with the costs covered by the Brazilian Unified Health System. Based on the mean number of patients who received chemotherapy for the first time in the outpatient clinic in the previous 12 months, the sample size was calculated by using G-power software, adopting a power of the test of 80% and a level of significance of 5%. Following Kang’s recommendations [37] for controlling type I (false positive) and type II (false negative) errors, the calculation was performed a priori, and the power of the effect was determined based on a previous study with a similar methodological design [10]. The result was a sample of 91 patients who were considered to have borderline representativeness, between moderate and high, according to systematic review [38]. 

### 2.2. Recruiting, Inclusion, and Exclusion Criteria

The inclusion criteria were malignity confirmed by means of a histopathological report and age of 18 years old or older. The exclusion criteria were as follows: individuals with a performance status greater than or equal to 3 on the Eastern Cooperative Oncology Group (ECOG) scale, which measures impairment of daily life activities [39]; and patients for whom no intravenous antineoplastic drugs were prescribed as part of the proposed therapeutic regimen.

The patients showed different types of malignity regarding diagnosis and staging. Recruiting occurred after the first individual nursing appointment, which was when fulfillment of the eligibility criteria was verified and the study objectives were presented. The proportion of patients who agreed to participate in the study was 98.91%; only one turned down the invitation, because he felt incapable of answering the questionnaire. Selection bias was reduced by adopting consecutive recruitment, and the effect of confounding variables was minimized by using statistical modeling. 

### 2.3. Instruments

A questionnaire for the collection of sociodemographic (age, sex, income, level of education, current professional activities, and religious beliefs) and clinical data (oncological diagnosis and treatment, performance status, anthropometry, and use of psychotropic drugs) was designed by the authors, with twenty open questions. To check difficulties in applying the instrument and the clarity of the questions, a pilot test was performed on a random sample of ten cancer patients. Analysis of the preliminary results revealed good understanding and usefulness of the tool. A second questionnaire containing three self-report scales was used to collect data related to pain, distress, and spiritual well-being. 

The Visual Analogue Scale (VAS), which is considered the gold standard for measuring pain intensity [40], was chosen as the evaluation method. The study followed the recommendations of clinical protocols and Brazilian therapeutic guidelines for pain control in cancer patients [41]. The speed of application, simplicity, and sensitivity make the use of this scale more advantageous in hospital and outpatient settings [42,43,44]. The pain VAS is a one-dimensional instrument, consisting of a continuous scale made up of a horizontal line 10 cm long, with each end of the line labeled with descriptors representing the extremes of pain intensity: 0 = no pain; 10 = worst pain [41,45,46]. In Brazil, despite being widely used and adapted to the country culture, few validation studies can be found [42,44,47,48,49,50]. In the present investigation, respondents were instructed to place a mark on the point (number) that represented their pain intensity level at that moment, considering 0–2 as mild pain, 3–7 as moderate pain, and 8–10 as severe pain.

In addition, the present study applied two instruments validated for use in the Brazilian population that showed satisfactory internal validity indexes, specificity, and sensitivity: the Distress Thermometer and Problem List for Patients, developed by the National Comprehensive Cancer Network [15] and validated in Brazil in 2009 [51]; and the Spiritual Well-Being Scale (SWBS), also validated in Brazil in 2009 [52]. 

The Distress Thermometer (DT) and the Problem List for Patients (PL) are components that make up an instrument based on self-reporting by patients with cancer to identify their level of distress and its possible causes over the previous week, including the day on which the evaluation is carried out. In the first part (DT), patients are asked to circle the number that best represents their level of distress on a visual analog scale that ranges from 0 (no distress) to 10 (extreme distress). In the second part (PL), patients are asked to indicate whether any of a list of issues associated with distress has been a problem for them, even if the issues are not associated with the diagnosis or treatment. The PL includes 35 problems separated into five categories (practical problems, family problems, emotional problems, spiritual/religious concerns, and physical problems). In this investigation, the scale showed a Cronbach’s alpha of 70%, with a sensitivity of 95.6% and specificity of 99% in determining distress by means of the DT [15].

The SWBS was added to the set of tools used in the present study to test the spiritual health of the patients and verify whether harm in this dimension was related to distress. This scale was designed in the 1980s by Paloutzian and Ellison [53]. The tool has two subscales and is made up of 10 items that refer to God or an absolute power (religious well-being, or RWB) and 10 items that concern the feeling of encountering something greater than themselves and commitment to a life purpose (existential well-being, or EWB). Before filling out the instrument, participants are instructed to interpret the word “God” in a way that is personally meaningful and consistent with their beliefs. Considered together, the two dimensions evaluate the individual’s general spiritual well-being, resulting from their relationship with God and their sense of direction and satisfaction in life (SWB) [53,54,55].

The 20 items must be answered by using a six-option Likert scale: strongly agree (6 points), moderately agree (5 points), agree (4 points), disagree (3 points), moderately disagree (2 points), and strongly disagree (1 point). The scale, which was designed to prevent response bias, was balanced in each dimension, with half the items being positive and half negative (7 to 10, 13 to 16, 18, and 20), with the latter being summed in an inverted way. To score the test, the numerical values for each response are added for each of the subscales. Both values for the subscales are then summed to reveal the total SWBS value. Scores range from 10 to 60 on the subscales and 20 to 120 on the SWBS. Higher scores reflect a higher perception of well-being, while lower scores reflect a lesser perception [53,54]. In the present study, the scale revealed a Cronbach’s alpha of 72%, with a sensitivity of 94% and specificity of 98%.

### 2.4. Data Collection Procedure

An expert in cancer nursing (author 1) approached the patients before the first day of chemotherapy (T1) to explain the study objectives and the procedures involved. The patients who agreed to participate were asked about their sociodemographic profile, and their answers were noted by the specialist in the first paper questionnaire. Then, patients were asked to fill out the second paper questionnaire containing the VAS, DT, PL, and SWBS. It was stressed that completion of the questionnaire required the participants to be honest, so the items chosen reflected their genuine feelings, thoughts, and behaviors. 

To control for observation bias, the participants filled out the instrument in a separate room. Social desirability bias, where respondents protect their belief system by reducing distress [56], was alleviated by asking participants to mark the SWBS items only after completing the DT. After the patients finished filling out the questionnaire, the researcher was available to answer possible questions about the instruments. Clinical data were obtained by consulting medical records. All items were answered by 91 patients, and it was not necessary to use statistical methods to correct missing data.

### 2.5. Statistical Analysis

Once the study variables were collected, a database was created with Excel software, and the information was confirmed by double-checking by Authors 1 and 7. Statistical analyses were carried out on R software version 4.4.1 (R Foundation, Vienna, Austria), with a level of significance of 5%. In descriptive analysis, nominal and ordinal qualitative variables were described as absolute numbers (*n*) and percentages (%), whereas quantitative variables were described by means, standard deviations (SD), minimum and maximum values, medians, and the inter quartile range (IQR).

The dependent variable of the study was presence or absence of distress. The participants were divided into two groups according to the cutoff established by Decat, Laros, and Araújo [51]: positive distress screening (DT ≥ 4) and negative distress screening (DT < 4). The independent variables were: (a) sociodemographic profile, namely sex, age, level of education, occupational activity, and income; (b) religiosity/spirituality, namely belonging to a religion, religious denomination, and scores on the Spiritual Well-Being Scale and its dimensions; and (c) clinical conditions, namely presence and intensity of pain, previous history of COVID-19, use of psychotropics, nutritional status, neoplasia topography, diagnosis year, staging, and concomitant treatment.

The Kolmogorov–Smirnov test was applied to verify the distribution normality. The relationship between the dependent variable distress and the qualitative variables was obtained by using Pearson’s chi-square test, the likelihood-ratio test, or Fisher’s exact test, depending on the best data fitting. Fisher’s exact test and the likelihood ratio are indicated when an analysis shows at least one of the cells had an expected value lower than five.

An analysis of the relationships between the variable distress (yes/no) and the quantitative continuous variables was carried out by applying Student’s *t*-test, analysis of variance (ANOVA), the Mann–Whitney U test, and the Kruskal–Wallis test, depending on the normality of the variables in question. 

Spearman’s correlation coefficient was used to evaluate the correlations between the DT, SWBS, and quantitative variables. This coefficient generates a number ranging from −1 to +1. The closer the result is to one of the extremes (−1 or 1), the greater the strength of the correlation. Values close to 0 imply weak or non-existent correlations. When the sign of the result is positive, it means that an increase in one variable implies an increase in the other variable. Negative values indicate that an increase in one variable implies a decrease in another. In all analyses, the value of *p* ≤ 0.05 was also considered.

Multiple regression analysis was applied to identify predictors associated with the outcome distress (yes/no). Multiple regression, by evaluating the association between a given variable and the outcome and adjusting its effect by the other independent variables included in the final regression model, promotes the control of potential confounding variables existing in the relationship between the outcome and the independent variables [57,58,59]. The Poisson regression (PR) with robust variance was calculated by resorting to the R software sandwich library. The model was chosen because of the high prevalence of the dependent variable (higher than 10%). In situations of frequent outcomes, an overestimated measurement of the association can be produced if the prevalence ratio is interpreted as an odds ratio. Once the odds ratio increases as the event becomes common, the literature suggests the use of alternative models, such as Cox regression, log-binomial regression, or Poisson regression with robust variance [57,58].

The multiple model was fitted by using the potential factors pointed out in univariate analyses as predictors. Based on the non-optimized stepwise forward method [58], only the variables that presented *p* ≤ 0.20 in univariate analyses were selected as the initial candidates to make up the final model. The candidates were the following variables: sex, age, and level of education; monthly income; pain intensity; staging; treatment type; performance status; concomitant treatment; previous use of psychotropics; RWB score; EWB score; and total SWBS score. The following candidate variables for the final model were continuous: age, pain intensity, RWB score; EWB score; and total SWBS score. It must be stressed that collinearity between candidate variables was tested before estimating the complete model, which led to formation of a set of models according to this result. Correlated variables were not part of this complete model. 

After inclusion and exclusion of the added variables, the significance of the interactions between those that remained was tested, following their order of influence on the outcome. Comparison of the models’ fitting was carried out by applying the Akaike information criterion (AIC) [58]. The choice of the final model took into account epidemiological and biological plausibility and a 5% statistical significance, with estimates of associations based on prevalence ratios (PRs) and their 95% confidence intervals [57,58]. 

## 3. Results

**Figure 1:**
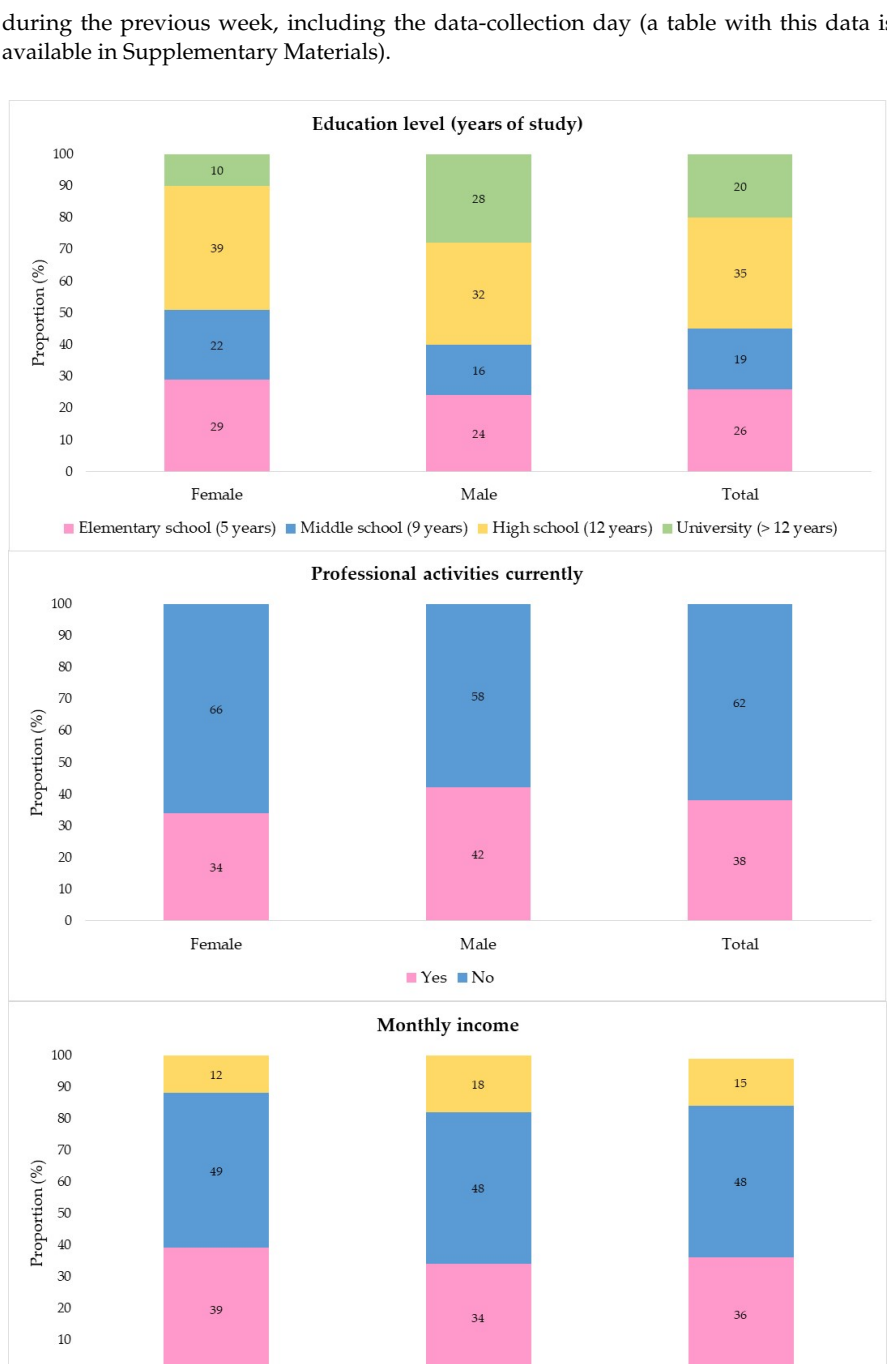
Distribution of sociodemographic variables of patients initiating chemotherapy during the COVID-19 pandemic, according to sex. Rio de Janeiro, RJ, Brazil, 2021 (*n* = 91).

The sample of the present study was 91 people. The average age was 55.4 years (SD 13.9), and the median was 58 years (20.5). Most participants were men (54.9%), did not have a professional activity when the interview occurred (61.5%), and had a monthly income between US $384 and US $576.00. As regards the level of education, 45.1% of the patients had up to middle school. The analysis by sex indicated that the women were older (60.0 [19.0] vs. 56.5 [12.4]) and had fewer years of schooling. More of the men were in professional activities at the time of data collection (42% vs. 34%), but they had a similar monthly income to the women (Figure 1; a table with this data is available in Supplementary Materials).

Over 90% stated that they belonged to a religion: 48.4% were Catholic, 38.5% were evangelical, and 6% were Umbanda or Spiritist. There was a higher prevalence of people with cancer in the gastrointestinal tract (39.6%), stage ≥ III (82.5%), and receiving palliative treatment (41.8%). Most patients were diagnosed with cancer in 2020 or 2021 (92.3%), showed good performance status (57.1%), and said that they did not use psychotropics (71.4%). Less than 10% reported infection with COVID-19 before data collection. When asked about the presence and intensity of pain, 79.1% chose the option “mild intensity” (Table 1).

**Figure 2:**
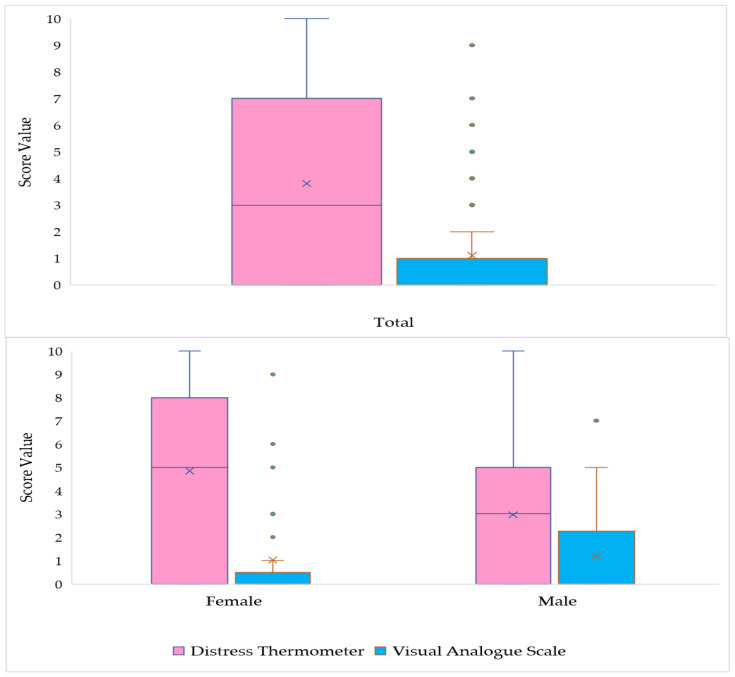
Boxplot of the distribution of the Visual Analogue Scale (VAS) score and Distress Thermometer (DT) score of patients initiating chemotherapy during the COVID-19 pandemic. Rio de Janeiro, RJ, Brazil, 2021 (*n* = 91).

The boxplot in Figure 2 shows that the VAS scores range from 0 to 9 in the sample, and are typically low. Scores higher than 2 on the scale are considered unusual events, (only six patients presented values above 5). The distribution is asymmetric to the right (positive asymmetry), demonstrating high concentration of low scores in the sample. A median equal to 0.0 (1.0) was observed, pain was reported by only 25.3% of patients, and women were more homogeneous concerning pain intensity than men (0.0 [0.0] vs. 0.0 [2.0]). In contrast to the VAS score, the DT score shows high variability, with no discrepant or atypical values. The median score is 3, women show greater variability in the intensity of distress than men, and 75% of the subjects showed values lower than or equal to 7. The distribution presents a slight asymmetry to the right, showing some behavior unpredictability. The prevalence analysis indicated that 49.5% of the patients experienced distress (DT ≥ 4) during the previous week, including the data-collection day (a table with this data is available in Supplementary Materials).

**Figure 3:**
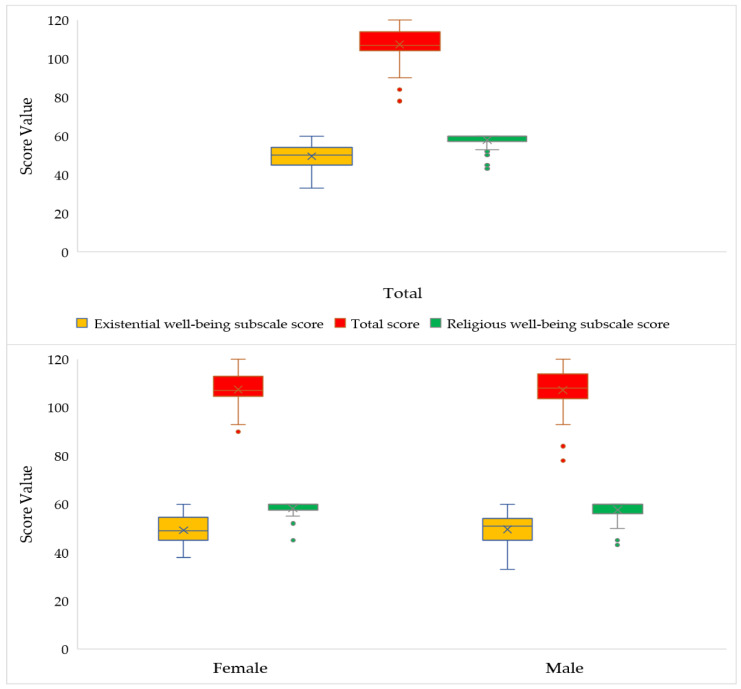
Boxplot of the distribution of the Religious Well-Being subscale score, Existential Well-Being subscale score, and total score on the Spiritual Well Being Scale of patients initiating chemotherapy during the COVID-19 pandemic. Rio de Janeiro, RJ, Brazil, 2021 (*n* = 91).

In the comparative boxplot in Figure 3, the SWBS and its subscales showed results with low-to-intermediate variability among the participants: 6.91% in the RWS dimension, 14.4% in the EWS dimension, and 8.50% in the total spiritual well-being score. The existential subscale presents a higher median score than the religious well-being scale, (50 vs. 60). The distribution of the religious well-being score presents strong asymmetry to the left (negative asymmetry), illustrating concentration of high values obtained with the subscale. The outliers in the figure show that scores lower than 53 are rare. As for the analysis of the point distribution of the total spiritual well-being scale, scores vary between 78 and 120, with values lower than 94 considered atypical in the sample. The median score is 107, with moderate asymmetry to the left (negative). The values of the spiritual well-being scale and its subscales are similar between men and women (a table with this data is available in Supplementary Materials).

**Figure 4:**
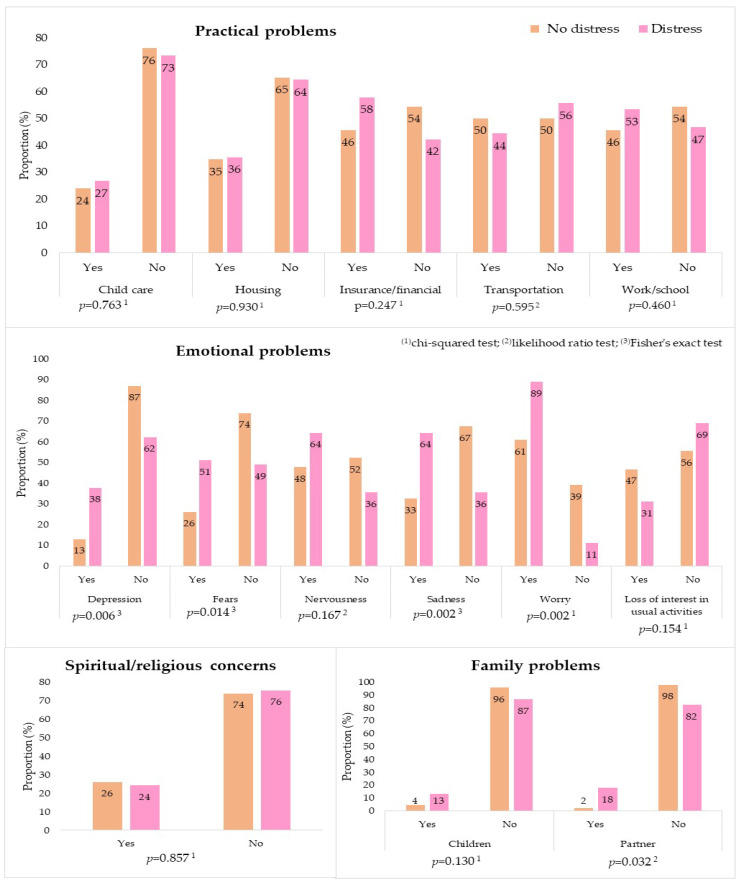
Distribution of practical problems, family problems, and spiritual/religious concerns, according to the Distress Thermometer and Problem List for Patients, between patients with and without distress who were initiating chemotherapy during the COVID-19 pandemic. Rio de Janeiro, Brazil, 2021, (*n* = 91).

**Figure 5:**
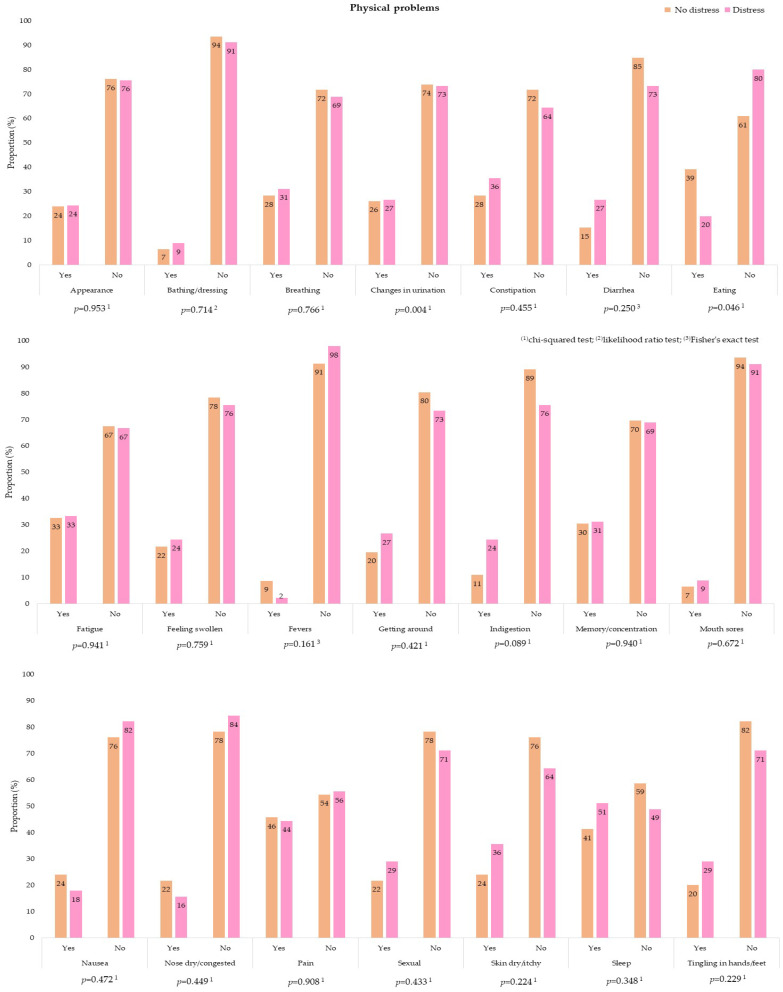
Distribution of physical problems, according to the Distress Thermometer and Problem List for Patients, between patients with and without distress who were initiating chemotherapy during the COVID-19 pandemic. Rio de Janeiro, Brazil, 2021, (*n* = 91).

Most participants reported not having the problems listed in the PL. For patients with distress, the most cited practical problems were related to health insurance/finances (57.8%) and work/school (53.3%). Family problems were rarely mentioned, and only 24.4% of the participants declared that they had difficulties in the sphere of religious and spiritual engagement (Figure 4). In contrast to what was found for previous categories, most emotional problems showed significant differences at the level of significance of 5% when patients with and without distress were compared. This analysis indicated a higher prevalence of concern (88.9% vs. 60.9%), nervousness (64.4% vs. 47.8%), sadness (64.4% vs. 32.6%), fear (51.1% vs. 26.09%), and depression (37.8% vs. 13.1%). In the physical category, four out of the 21 listed items were chosen more often by patients with distress: sleep (51.1%), pain (44.4%), dry skin (35.6%), and fatigue (33.3%). However, there was no significant difference between the two groups (Figure 5). A table with this data is available in Supplementary Materials.

The results of comparative analysis showed statistically significant relationships between positive screening for distress and the variables sex, age, and diagnosis year, with a higher prevalence among women (*p* = 0.021), younger patients (*p* = 0.027), and those who received their diagnosis during the COVID-19 pandemic (*p* = 0.042), especially in 2020 (*p* = 0.013). Concerning the SWBS, the EWB subscale of patients who experienced distress showed the lowest mean and median (*p* = 0.031) (Table 2). 

A weak negative correlation was found between the score on the DT and the variables age (0.029), EWB score (0.005), and SWB score (0.013). In other words, the higher the age and the scores on EWB and SWB, the lower the value assigned in the DT. Similarly, a weak negative correlation was identified between the variable pain and EWB score (0.004) and SWB score (0.002), indicating a lower pain intensity among those with higher levels of existential and spiritual well-being (Table 3).

After fitting the multiple model, the variables that remained associated with distress were age, sex, and EWB score. Confirming the results of bivariate analysis, we see that there was a lower prevalence of distress in men, older patients, and those with higher scores in the EWB. Compared to men, women showed a 1.7 higher chance of reporting stress. Additionally, a one-year reduction in age increased the probability of experiencing distress by 1.52% (PR = 0.985), whereas a one-point reduction in the EWB scale increased the distress prevalence by 3% (PR = 1.030) (Table 4). 

Table 5 shows descriptive statistical analysis of all questions related to the SWBS. The SWBS items that stood out belonged to the religious domain. The highest response frequency in ”Strongly agree” was found for the following items: 1—My relationship with God contributes to my sense of well-being (89); 4—My relationship with God helps me not to feel lonely (89); 5—I believe that God is concerned about my problems (87); and 3—I feel most fulfilled when I am in close communion with God (83). In the EWB subscale, the items that stood out were as follows: 19—I believe there is a real purpose for my life (84); and 14I—(inverted item) I do not enjoy much about life (81). 

## 4. Discussion

In the present study, screening showed a distress prevalence of 49.5%, which is higher than that found before the pandemic in the Netherlands (34.5%) [25] and Korea (33.6%) [60], and in a database with more than 9000 registered profiles (35.1%) [24]. The prevalence was also different from that found in China in patients who were hospitalized to receive treatment (22.1%) [36], but similar to the rate found in countries such as India (44.5%) [61] and United States (42.0%), where patients who had just been diagnosed with cancer were at a higher risk of experiencing distress [62]. Regarding the average score on the DT (3.81), a quasi-experimental study reported a similar value for a group of patients who were about to receive cancer treatment (3.72). According to the authors, the psychological distress associated with chemotherapy can be reduced by psychoeducational intervention (average score of 2.92 after intervention), which highlighted the importance of the DT as a screening instrument and for follow-up on therapeutic measures [63].

In Brazil, the psychological distress status in this population seems to have been made worse by measures to control COVID-19, because they caused increased wait times to initiate cancer treatment [3,64,65,66]. A Brazilian study indicated a reduction of 57.4% in the number of patients initiating cancer treatment, and of 27.5% in the number of patients receiving intravenous systemic treatment [3]. This context may explain the high prevalence of distress found in the present study, especially because data collection occurred over the most serious phase of the pandemic in Brazil. During this period, the country recorded the highest infection, hospitalization, and death rates associated with COVID-19, and the Brazilian health system collapsed [67]. This suspicion is supported by the findings of Brazilian authors who found, in 2018, low levels of distress in patients with indications for antineoplastic chemotherapy [10], as well as in a study that compared quality of life before and during the pandemic. They demonstrated that the rescheduling of treatment cycles in Brazil induced emotional imbalance due to the disturbing nature of waiting for treatment [68]. In this regard, difficulties in accessing diagnostic services, specialized therapy, and psychological support may have contributed to a greater prevalence and severity of distress in the pandemic period.

We also believe that the way the news was disseminated in Brazil—constantly stressing the information that patients with cancer were a risk group for infection, complications, and death from COVID-19—exacerbated psychological stress [65]. Additionally, the fact that some hospital users live far from the chemotherapy clinics, have low purchasing power, and consequently depend on public transportation may have contributed to increasing levels of distress, because these patients may have feared getting infected on the way to the hospital. However, the results did not show a correlation between distress and previous history of infection with SARS-CoV-2. Despite the higher risks of serious complications and stigmatization, myths about immunity and fake news about the real epidemiological situation [69,70] may have resulted in a false feeling of safety, making people less worried about being infected.

Other authors have discussed how diagnostic inaccuracy, fear that the disease will progress, and movement among several health units in search of care bring about the perception of unnecessary delay, culminating in feelings of anger, indignation, and legal implications, such as medical malpractice claims and payment of compensation [27,71,72]. This body of evidence serves as a warning to leaders about the need, not only to stop virus dissemination, but also to develop strategies that ensure that patients have the right to complete their diagnostic investigation, get timely cancer treatment, receive psychosocial care, and be submitted to rigorous COVID-19 screening. 

Relational analysis of the PL items and the variable distress pointed out higher prevalence with statistical significance for emotional problems, especially concern (88.9%), sadness (64.4%), and fear (51.1%). This result is consistent with a study that distinguished the emotional component as dominant in the experience of distress [73]. For the authors, the importance of the category is such that distress patients could be diagnosed by only ticking concern or depression in the PL. Despite the high proportion of patients in advanced stages of cancer, and indications for palliative treatment, physical problems were rarely reported, except for pain (44.4%) and difficulty sleeping (51.1%); however, they were not associated with distress. Positive associations were found only for urinary (26.7%) and eating disorders (20.0%). These results differed from those of a study carried out with Indian patients with a diagnosis of head and neck cancer before chemotherapy was initiated [61]. In this case, the main problems mentioned by the patients were related to pain, changes in appearance, and financial concerns. These differences may be explained by the diagnostic profile. Patients with head and neck cancer are more affected by mutilating treatments, speech dysfunctions, facial disfigurement, and adverse socioeconomic conditions, which usually cause the risk factors for the disease to be higher [36,74].

It is important to emphasize that before the first treatment cycle, the emotional disorders cited in the PL can predominate and result in more distress. Qualitative [27] and quantitative [36,38] studies have explained this result. It is postulated that the distress state, critical at this point, is characterized by the shock caused by the diagnosis, sadness due to loss of hope, fear of the effects of the cytotoxic therapy, and concerns about an uncertain future. These pessimistic expectations are likely to have become more acute during the critical phase of the COVID-19 pandemic in Brazil, the period in which the present study was carried out.

Evaluation of the variables associated with distress (DT ≥ 4) showed that this outcome was significantly more prevalent in women, younger people, and patients with lower scores on the EWB dimension. However, it has to be stressed that most variables in the socioeconomic and clinical profile were not associated with distress in the sample of patients initiating chemotherapy. Even variables such as treatment modality, pain, and income lost their significance when included in the regression model. Contrary to the conclusions of other studies [75,76,77,78], our results suggest that adverse prognostic factors do not explain psychological distress better than variables such as age, sex, and scores on the EWB, which can be taken as a sign of good spiritual health. Previous evidence also showed that cancer stage and treatment objectives were not associated with greater distress when the situation of delayed diagnosis is taken into account [79]. 

In contrast to a study that evaluated cancer patients in Korea [80], we did not find an association between performance status (PS) and distress. The Korean authors found higher levels of distress in patients with worse PS, especially in younger patients. Higher functional performance scores usually reflect better quality of life, function, and self-care capacity. This indicator is widely used to select patients suitable for more aggressive treatments, such as surgery and radiotherapy [81]. It is possible that the late effects of such therapies negatively affected the group of patients with better performance, balancing the emotional consequences of a decline in physical abilities. Despite the proven risk of depression in patients with impaired performance status [82], some authors suggest the absence of a cause-and-effect relationship, and even an influence of anxiety and depression on the worsening of functional status [81].

Brazilian studies on distress in cancer patients have indicated that even more important than the physical condition of the patients are the spiritual resources they have at their disposal and the way they interpret and reevaluate the experience of living with the disease [10,27]. According to this view and Folkman’s theoretical framework [83], people with incurable cancer can have their distress counterbalanced by hope, a construct that is considered subjective, dynamic, and contextual. As claimed by this author, by dealing with the uncertainty of the diagnosis, people can customize their success probabilities through personal and spiritual characteristics, regardless of the established medical risk and unfavorable prognostic factors. 

These assumptions seem to be confirmed by the present study, which showed high religious, existential, and spiritual well-being scores in a predominantly religious sample (93.4%). Similar evidence was found by Rowold [84], whose study demonstrated that spiritual well-being was a predictor variable of happiness and psychological well-being, and was associated with lower levels of stress. Rawdin et al. [85] concluded that hope is not related to variables such as age, sex, or cancer, but to spiritual well-being, which overcomes any association with pain or disease severity. Nevertheless, we caution that the lack of association between clinical factors and distress found in our study may be related to the characteristics of the sample, including the low number of patients in the curative and adjuvant treatment groups.

The present study also found significant but weak negative correlations between the DT scores and EWB and SWB scores, and between the latter and pain intensity. These associations have been discussed in previous studies with cancer patients [86] and patients with spinal injuries [87], which have reinforced the thesis that reduced spirituality scores can aggravate the physical experience of pain and other symptoms. As regards distress measured by the DT and its bidirectional and, sometimes, complementary association with pain, the literature indicates that this physical symptom can expand the self constructively. If the individual does not attribute a threatening meaning to it, they do not believe themselves to be in danger, and, consequently, no distress is perceived [88]. Therefore, despite its known impact on anxiety and depression, the direction and effects of this relationship may be more complex. Still, it is surprising that the present study did not find a correlation between these two variables, contrary to what was shown in a study by Li et al. [77]. We speculate that the reduced proportion of patients with pain and the presence of other sources of distress in people without pain affected the results of our study. 

Concerning the influence of sociodemographic variables, only female sex and lower age were associated with distress. This finding is consistent with those obtained for other populations of cancer patients in studies in Brazil [89], Japan [90], and Poland [91], with the latter carried out in the COVID-19 pandemic context. Data from the literature have shown that women with cancer tend to report more distress, and that changes in self-image and psychosocial and sexual disorders, as well as family conflicts, can affect them more intensely [85,92]. A systematic review and a meta-analysis have indicated that, in the general population and in the COVID-19 pandemic period, women and young adults had higher chances of experiencing distress because of higher levels of depression, anxiety, and post-traumatic stress disorder [1,6,93]. These factors were also confirmed in outpatients with blood cancer during the first phase of restrictions in hospital care in Italy as a consequence of the pandemic [34]. Women’s vulnerability was also observed during the COVID-19 quarantine, and it was also present in the outbreak of other infectious diseases [94] and in people with diagnoses other than cancer [95]. 

In the younger population, greater access to electronic information and social media, concern with their careers, and multiple obligations, such as working and being responsible for the care of other people, are probable reasons for the higher load of psychological stress in this age group [1,6]. In this phase, life trajectories truncated by cancer force them to replace long-term goals with short-term goals. Therefore, it is plausible that the multiple required trips to the hospital, such as coming in for blood sampling, appointments, and therapeutic interventions, caused more distress in the young people in the sample of the present study, who may have faced difficulties with carrying on with their education, professional activities, and social relationships. Our results suggested that the factors age and sex are probably involved in the perception of distress. Taken in combination, these findings reiterate the urgent need to pay attention to subgroups that are more deprived of psychosocial support.

The variables level of education and income were included in the present study because of the hypothesis that there is a higher number of stress predictors among people with low level of education and low income. Authors who have supported this association have cited the following stressors: little ability to understand and manage symptoms; concern with family expenses; insufficient psychosocial support; loans; and fewer resources for dealing with adverse effects, such as being unable to buy more powerful antiemetics [96,97,98]. Nevertheless, our results did not confirm these assumptions. It can be presumed that, because the study location was a public institution that provides medication, food, and accommodation on chemotherapy days, the deleterious financial effects were reduced. Regarding the level of education, if a lower level of formal instruction may predispose patients to underemployment and work overload, a higher educational level may be associated with awareness of the risks of the disease and deep understanding of the morbidity process, leading to more intense psychic distress. 

Concerning SWBS and its subscales, the scores obtained in the study did not show an association with age, as reported by other authors [99,100]. The results differed from those of a Japanese study that indicated higher levels of spiritual well-being in elderly people. As reported by the researchers, younger patients tend to be more challenged when it comes to finding a meaning for their lives after receiving the diagnosis, a condition more prevalent in the elderly population [101]. It is believed that thoughts about the reasons for the development of the disease can affect younger patients more intensely. Experts in spirituality emphasize that elderly people may accept finitude better if a successful aging process makes them look at adversity from a mature spiritual perspective [102]. However, cultural differences between samples and the use of different instruments to evaluate spiritual well-being may be involved in the divergences. 

Given the scarce literature on distress in patients initiating chemotherapy [61,103], few studies have assessed the role of spiritual well-being and its association with a diverse set of variables in this population [6,104], which has limited the scope of the discussion. Nevertheless, the participants had a higher average spiritual well-being score (107.93) in comparison with patients under chemotherapy in Portugal [104] and Brazilians with heart diseases evaluated before the COVID-19 pandemic [105]. Already in the pandemic situation, a study in Italy found lower levels of spiritual well-being compared to the prepandemic situation. Similar to the present study, women’s mental health was more affected. The authors suggested that the foundations of good spiritual health were shaken with the closing of religious institutions and social distancing [33]. Compensatory strategies implemented in Brazil to face these limitations, such as offering religious services, meetings, and worships online [106], may have lessened the deleterious effects of the situation. 

Analysis of the items that received the highest scores on the SWBS suggested that the challenging nature of the diagnosis and the crisis triggered by COVID-19 caused a more active search for spiritual and religious connections. An example of this is the creation of free phone lines for direct spiritual support in Brazil [107] and initiatives in favor of collective care, empathy, and solidarity [108]. Although the importance of spiritual well-being in the psychological adjustment of people living with cancer is recognized, understanding the relationship between this construct and distress remains a challenge for health professionals.

It is necessary to emphasize some points that have to be handled with caution in making inferences and conclusions based on the data from this study:

1. The terms “distress”, “spirituality/religiosity”, and “well-being” may have different meanings for people from different cultures, whether they are patients or health professionals.
2. Distress and spirituality are not directly observable phenomena and are especially sensitive to the presence of evaluators [55,56]. Additionally, there are no clear signs related to them that lead to a definitive interpretation [109].
3. For the outcome distress, multiple psychic determinants show interdependence and a mutable nature, varying over a short period [28]. Because their effects manifest in a global and integrated manner, the way people perceive, feel, or behave when confronted with adversity makes it impossible to establish inferences based on analyses with only one predictor.
4. In a country as religious as Brazil [110], admitting distress can be considered lack of faith, especially for some followers of Christianity. Therefore, awareness that their spiritual beliefs would be put to the test may have influenced the participants’ answers.
5. Given the complexity and difficulty of distinguishing the constructs religiosity and spirituality [110], identifying the role played by both in the perception of distress becomes important.

It was expected that religious well-being would work as a dimension contrary to distress because, despite their differences, both phenomena encompass, in their essence, emotions that can act as mutual counter-regulators. The results indicated that feelings of spiritual connection and a strong sense of life purpose can outweigh the perception of religious well-being in patients with a tendency to report less distress. In addition, it is possible that many advantages of religious well-being, such as a sense of belonging to a group that shares social relationships, have been compromised during the COVID-19 pandemic. Spirituality, in turn, construed as a more sacred dimension of people and not limited to rituals or institutions, played a more intense role in the process of coping with distress. We believe that a deep reevaluation of who they were before and after cancer offered the participants the possibility, not only of experiencing personal growth, but also of developing a new sense of being [27]. This may have motivated patients with higher existential well-being scores to transcend distress. 

This hypothesis is supported by studies that have identified spiritual coping styles that are mostly positive among cancer patients [20,111]. Results from the current study suggest that spiritual problems (24%) are less common than previously thought for individuals diagnosed with distress, as demonstrated by a multicenter study involving an ethnically diverse sample [112]. Similarly, a DT validity study in Saudi Arabia deduced that the relationship with God is little affected by distress after controlling for confounding variables [113]. In Turkey, equally high levels of distress and positive coping were found among chemotherapy patients. However, those with better coping patterns suffered less from treatment [20,111]. Turkish Muslims [111] and Brazilian Christians [20,114] share the belief that, in situations of serious illness, they should be guided by religion. Both religious cultures embody the notion that distress is part of life, and without it, they could not approach God with sturdy faith, remaining immersed in their own desires and clinging to meaningless concerns [111,114,115]. The intensity and importance of this approach with God were strongly evidenced in the scores assigned to items 1, 3, and 4 of Table 5. The multiple model showed that the spiritual component is an important predictor of distress, but the religious dimension, in general, can provide answers to existential questions. Ultimately, the connection with God, for some religions, leads to the experience of a deep disposition to feel one with and part of a greater purpose in life. In this relationship, subjects access spirituality by questioning themselves about meaning, while in religiosity, they can find a way to solve the enigmas of an incomprehensible destiny [32]. In the tension of stress and threat to the biopsychosocial integrity of their being, there is a favorable field for the development of spiritual reflections, capable of moving life forward by finding new goals. Consequently, stress constitutes a means by which latent spiritual forces are brought into action. Such forces can break through inner protective layers that prevent a more meaningful contact with who we are, what we do, and why we do it, when we are not experiencing distress. 

Lastly, the results confirmed that, in the population of this study, high levels of religiosity/spirituality are expressed when faced with catastrophic events, such as the beginning of cancer treatment in a pandemic context. We hypothesize that spirituality may be used as a resource to turn distress into resilience, thus strengthening and improving collective coping standards. Although correlations with distress were not found at the expected level, it is believed that this resource may provide strategies to lessen distress, as indicated in the measures of association. New studies that address the religious and spiritual dimensions of distress can contribute to this direction. Therefore, we emphasize the need to design new longitudinal studies that can capture dynamic nuances involved in the processes of distress transformation and spiritual well-being, and examine the role played by religiosity and spirituality in these processes. Cohort studies will make it possible to investigate possible oscillations during the post-diagnosis, active treatment, and exclusive palliative care periods.

One of the strengths of the present study was analyzing an extensive set of variables and their implications for the outcome of distress in patients initiating chemotherapy. The psychological impacts resulting from the delay of the beginning of cancer treatment caused by the COVID-19 pandemic, still poorly understood, were widely discussed. Analysis originating in correlations based on a single measurement and, consequently, subject to reverse causality can be considered a limitation of the present study, because this type of analysis precludes conclusive inferences about the strength and directionality of the causal relationship between spiritual well-being and distress. Additionally, generalization to other contexts should be made with caution, due to the size of our sample and the fact that the study setting is a public hospital, which may affect the time to start treatment and perception of psychological distress.

## 5. Conclusions

A high prevalence of distress was found in a sample of patients initiating chemotherapy in a city in the southeast region of Brazil. Fear, pain, and distress challenged religious beliefs, but spiritual well-being remained high despite the atmosphere of uncertainty about continuity of life. Lower age and female sex were associated with an increased risk of distress, whereas statistical modeling showed that existential well-being worked as a protective factor. A high proportion of the patients was at advanced stages of cancer and had received indications for palliative chemotherapy. In this group, emotional problems were more relevant than physical issues. Among patients with positive distress screening, the most significantly prevalent problems were concern, sadness, and fear. 

After control of confounding variables, distress was not associated with the expected clinical or sociodemographic factors, such as cancer stage, nutritional status, performance status, pain, treatment modality, and income. Therefore, arbitrarily presuming that patients in a disadvantaged clinical and socioeconomic situation are more likely to report distress can lead to mistakes in risk stratification and care planning. 

Weak negative correlations were observed between pain intensity and existential and spiritual well-being levels, confirming the effects of spirituality on the physical dimension of distress. A negative correlation was also found between distress scores and levels of existential well-being. Our analysis of risk factors involved in distress in patients initiating chemotherapy during the COVID-19 pandemic allowed for the identification of subgroups more vulnerable to mental-health problems. 

### 5.1. Implications for Clinical Practice

Screening patients with a high risk of developing distress during the COVID-19 pandemic is fundamental for planning and carrying out comprehensive care. Identifying risk factors can support therapeutic plans and actions in future outbreaks of this and other diseases. The reason is that risk stratification gives health teams the advantage of being able to get ahead of the development of more serious adaptation disorders. In particular, health professionals who work in cancer outpatient units occupy a position that allows them to identify patients with cancer who are experiencing distress and whose negative coping patterns may lead to loss of hope and difficulty finding a new purpose in life amidst chaos. Helping those with poorer spiritual well-being scores express their distress and manage fear is the first step toward strengthening their self-confidence and leading them to recover connections with themselves and with life. In the case of patients who have expressed higher levels of spirituality, professionals can channel this strength toward the formation of a shield to protect against distress.

### 5.2. What This Article Contributes to Public Health

The results are a challenge to leaders, because they indicated that, ideally, distress relief would involve the implementation of public health programs that are capable of integrating spiritual interventions into cancer care and recognition of the specific needs of women, who often experience overload because society imposes on them the role of main care provider. Considering the emotional imbalances triggered by cancer, the destruction of their dreams, and career interruptions in a period in which advancement is expected, another difficult task arises for policymakers: restoring in young adults with cancer a sense of control, autonomy, and freedom to allow them to resume some of their activities while they carry on with their treatment. Moving in this direction, and providing the best possible care for all, regardless of socioeconomic conditions, religious values, and gender, must be an essential attribute of any health service. However, it is necessary to develop subgroup-sensitive strategies to help people who are made vulnerable, with the objective of relieving the stress inherent in the factors that put these people in a disadvantageous situation. By predicting positive results brought about by spirituality, the present study also encourages the following actions in public health: training of specialists in spiritual care; and recognition of the available resources, including knowledge of the impact of spiritual interventions on local cultures and how they can induce healthier behaviors. In the future, managers will be able to not just associate cancer with risk factors, such as smoking. With the same confidence that health professionals warn the population about these risks, it will be possible to tell patients with cancer that, if they are spiritualized enough, they will be able to deal with their emotions better and protect themselves, at least partially, from the harmful effects of distress and the load of existential and psychic symptoms caused by it.

## Figures and Tables

**Table 1:** Distribution of clinical variables of patients initiating chemotherapy during the COVID-19 pandemic. Rio de Janeiro, RJ, Brazil, 2021 (*n* = 91).

Variables	*n*	%
COVID-19 infection		
Yes	7	7.7
No	84	92.3
Nutritional status		
Low weight	9	9.9
Eutrophic	34	37.4
Overweight	32	35.2
Obesity	16	17.6
Primary tumor location		
Genitourinary	9	9.9
Gastrointestinal	36	39.6
Hematologic	18	19.8
Lung	7	7.7
Head and neck	15	16.5
Central nervous system	1	1.1
Liver, pancreas, and biliary tract	3	3.3
Unknown primary site	2	2.2
Cancer staging		
I	2	2.2
II	11	12.1
III	37	40.7
IV	38	41.8
Not defined	3	3.3
Performance status		
PS-0	16	17.6
PS-1	52	57.1
PS-2	23	25.3
Type of treatment		
Curative	16	17.6
Adjuvant	11	12.1
Neoadjuvant	26	28.6
Palliative	38	41.8
Year of diagnosis		
2013	1	1.1
2017	2	2.2
2018	2	2.2
2019	2	2.2
2020	48	52.7
2021	36	39.6
Diagnosis during the pandemic		
No	7	7.7
Yes	84	92.3
Concurrent treatment		
No	70	76.9
Hormone therapy	4	4.4
Radiotherapy	17	18.7
Use of psychotropic drugs		
Yes	26	28.6
No	65	71.4

**Table 2:** Distribution of sociodemographic, spiritual, and clinical variables and significance of the differences between patients with and without distress who were initiating chemotherapy during the COVID-19 pandemic. Rio de Janeiro, Brazil, 2021, (*n* = 91).

Variables	No Distress		Distress		*p*-Value
	*n*	%	*n*	%	
Sociodemographic					
	Median	IQR	Median	IQR	
Age (years)	60.0	3.00	58.00	2	0.027 ^(2)^
Sex					
Female	15	32.6	26	57.8	0.021 ^(3)^*
Male	31	67.4	19	42.2	
Education level (years of study)					
Elementary school (5 years)	15	32.6	9	20.0	0.335 ^(3)^
Middle school (9 years)	10	21.7	7	15.6	
High school (12 years)	13	28.3	19	42.2	
University (>12 years)	8	17.4	10	22.2	
Performs professional activities currently					
Yes	16	34.8	19	42.2	0.304 ^(4)^
No	30	65.2	26	57.8	
Monthly income					
≤$192	19	41.3	14	31.1	0.302 ^(1)^
$384–$576	24	52.2	24	53.3	
≥$768	3	6.5	7	15.6	
Religiousness					
Religion					
Yes	43	93.5	42	93.3	0.650 ^(3)^
No	3	6.5	3	6.7	
Type of religion					
No religion	3	6.5	3	6.7	0.865 ^(3)^
Catholic	24	52.2	20	44.4	
Evangelical	16	34.8	19	42.2	
Umbanda	1	2.2	2	4.4	
Spiritist	2	4.4	1	2.2	
Clinical					
COVID-19 infection					
Yes	4	8.7	3	6.7	0.512 ^(3)^
No	42	91.3	42	93.3	
Nutritional status					
Low weight	7	15.2	2	4.4	0.178 ^(1)^
Eutrophic	19	41.3	15	33.3	
Overweight	14	30.4	18	40.0	
Obesity	6	13.0	10	22.2	
Primary tumor location					
Genitourinary	4	8.7	5	11.1	0.544 ^(1)^
Gastrointestinal	17	37.0	19	42.2	
Hematologic	9	19.6	9	20.0	
Lung	4	8.7	3	6.7	
Head and neck	8	17.4	7	15.6	
Central nervous system	0	0.0	1	2.2	
Liver, pancreas, and biliary tract	3	6.5	0	0.0	
Unknown primary site	1	2.2	1	2.2	
Cancer staging					
I	0	0.0	2	4.4	0.297 ^(1)^
II	4	8.7	7	15.6	
III	18	39.1	19	42.2	
IV	2	4.4	1	2.2	
Not defined	22	47.8	16	35.6	
Performance status					
PS-0	5	10.9	11	24.4	0.167 ^(1)^
PS-1	30	65.2	22	48.9	
PS-2	11	23.9	12	26.7	
Type of treatment					
Curative	7	15.2	9	20.0	0.057 ^(1)^
Adjuvant	2	4.4	9	20.0	
Neoadjuvant	17	37.0	9	20.0	
Palliative	20	43.5	18	40.0	
Year of diagnosis					
2013	0	0.0	1	2.2	0.013 ^(1)^*
2017	2	4.4	0	0.0	
2018	2	4.4	0	0.0	
2019	2	4.4	0	0.0	
2020	18	39.1	30	66.7	
2021	22	47.8	14	31.1	
Diagnosis during the pandemic					
Yes	40	87.0	44	97.8	0.042 ^(1)^*
No	6	13.0	1	2.2	
Concurrent treatment					
No	31	67.4	39	86.7	0.085 ^(1)^
Hormone therapy	3	6.5	1	2.2	
Radiotherapy	12	26.1	5	11.1	
Use of psychotropic drugs					
Yes	14	30.4	12	26.7	0.140 ^(1)^
No	32	69.6	33	73.3	
Visual Analogue Scale (VAS)					
Pain					
Yes	10	21.7	13	28.9	0.294 ^(3)^
No	36	78.3	32	71.1	
Pain intensity					
Mild (0–2)	37	80.4	35	77.8	0.489 ^(1)^
Moderate (3–7)	9	19.6	9	20.0	
Severe (8–10)	0	0.0	1	2.2	
VAS score	Median	IQR	Median	IQR	
	0.00	0.00	0.00	2.00	0.280 ^(2)^
Spiritual Well-Being Scale					
	Median	IQR	Median	IQR	
Religious well-being subscale score	60	0.74	60	1.0	0.628 ^(5)^
Existential well-being subscale score	51.5	2.2	60	3.0	0.031 ^(5)^*
Spiritual well-being (total score)	109.5	2.9	105.0	11	0.057 ^(2)^

SD, standard deviation; ^(1)^ likelihood ratio test; ^(2)^ Mann–Whitney U test; ^(3)^ Fisher’s exact test; ^(4)^ chi-squared test; ^(5)^ Kruskal–Wallis test; * *p ≤* 0.05 (statistically significant).

**Table 3:** Variables correlated with the scores obtained in the Distress Thermometer and the Spiritual Well-Being Scale for patients who were initiating chemotherapy over the COVID-19 pandemic. Rio de Janeiro, Brazil, 2021 (*n* = 91).

Variables	r	*p*-Value ^(1)^
Distress Thermometer		
Age	−0.228	0.029 *
Pain	0.166	0.115
Religious well-being	0.022	0.834
Existential well-being	−0.292	0.005 *
Spiritual well-being	−0.259	0.013 *
Spiritual Well-Being Scale		
Religious well-being subscale		
Age	0.15	0.887
Pain	−0.85	0.418
Existential well-being subscale		
Age	0.128	0.227
Pain	−0.2995	0.004 *
Spiritual well-being		
Age	0.115	0.278
Pain	−0.333	0.002 *

^(1)^ Spearman’s correlation coefficient; * *p ≤* 0.05 (statistically significant).

**Table 4:** Multiple analysis by means of Poisson regression (PR) with robust variance for the variables associated with distress in patients initiating chemotherapy during the COVID-19 pandemic. Rio de Janeiro, Brazil, 2021 (*n* = 91).

Variable	Unadjusted PR (95% CI)	*p*-Value	Adjusted PR (95% CI)	*p*-Value
Age	0.984 (0.971–0.997)	0.020	0.985 (0.973–0.996)	0.012 *
Sex				
Female	1			
Male	0.599 (0.392–0.9152)	0.018	0.588 (0.392–0.881)	0.010 *
Existential well-being	0.970 (0.944–0.997)	0.030	0.971 (0.946–0.996)	0.024 *

CI, confidence interval; * *p ≤* 0.05 (statistically significant).

**Table 5:** Descriptive analysis of the answers for items on the Spiritual Well-Being Scale given by patients initiating chemotherapy during the COVID-19 pandemic. Rio de Janeiro, Brazil, 2021 (*n* = 91).

Items	Likert Scale					
	1	2	3	4	5	6
1. My relationship with God contributes to my sense of well-being	0	0	0	1	1	89
2. I believe that God loves me and cares about me	1	0	0	2	1	87
3. I feel most fulfilled when I am in close communion with God	0	0	0	0	8	83
4. My relationship with God helps me not to feel lonely	0	0	1	1	1	89
5. I believe that God is concerned about my problems	0	0	1	1	2	87
6. I have a personally meaningful relationship with God	1	0	6	1	8	75
7I. I do not have a personally satisfying relationship with God	6	1	2	2	1	79
8I. I do not find much satisfaction in private prayer with God	2	2	0	4	4	79
9I. I do not get much personal strength and support from my God	4	0	1	1	2	83
10I. I believe that God is impersonal and not interested in my daily situations	3	0	2	1	2	83
11. I feel a sense of well-being about the direction my life is headed in	10	1	20	5	9	46
12. I feel very fulfilled and satisfied with life	6	2	19	8	14	42
13I. Life does not have much meaning	5	1	1	5	2	77
14I. I do not enjoy much about life	3	2	1	2	2	81
15I. I feel good about my future	10	7	5	13	3	53
16I. I feel that life is full of conflict and unhappiness	15	7	7	9	6	47
17. I feel that life is a positive experience	2	1	2	2	7	77
18I. I feel unsettled about my future	33	13	6	13	1	25
19. I believe there is some real purpose for my life	0	0	1	1	5	84
20I. I do not know who I am, where I came from, or where I am going	58	3	8	1	3	18

I: indicates the negative items, whose score was inverted. The religious well-being subscale is the first half (from item 1 to 10), and the existential well-being subscale is the second half (from item 11 to 20).

## Data Availability

The data presented in this study are available on request from the corresponding author.

## Supplementary Materials

The following are available online at https://www.mdpi.com/article/10.3390/ijerph182413200/s1, Table S1: Distribution of sociodemographic, spiritual, and clinical variables of patients starting chemotherapy in the pandemic. Rio de Janeiro, RJ, Brazil, 2021 (*n* = 91), Table S2: Distribution of problems according to the Distress Thermometer and Problem List for Patients classification, and significance of differences between patients with and without distress starting chemotherapy in the pandemic. Rio de Janeiro, Brazil, 2021, (*n* = 91).
