# Supplementary material for: Distress and Spiritual Well-Being in Brazilian Patients Initiating Chemotherapy during the COVID-19 Pandemic—A Cross-Sectional Study
Source: Int J Environ Res Public Health. 2021 Dec 15;18(24):13200. doi: 10.3390/ijerph182413200 (PMC8702099; doi:10.3390/ijerph182413200)
Supplement: Supplementary file 1 [file ijerph-18-13200-s001.zip › ijerph-1447578-supplementary.pdf]

Table S1. Distribution of sociodemographic, spiritual, and clinical variables of patients starting chemotherapy in the pandemic. Rio de Janeiro, RJ, Brazil, 2021 (n = 91)

**Variables**	**n**	**%**
Sociodemographic		
Age group (years)		
20–39	15	16.5
40–59	35	38.5
60 or more	41	45.1
Age (Years)		
Mean (standard deviation)	55.4 (13.9)	
Minimum (maximum)	21 (86)	
Sex		
Female	41	45.1
Male	50	54.9
Education level (years of study)		
Elementary school (5 years)	24	26.4
Middle school (9 years)	17	18.7
High school (12 years)	32	35.2
University (>12 years)	18	19.8
Performs professional activities currently		
Yes	35	38.5
No	56	61.5
Monthly income		
≤ $192	33	36.3
$384-$576	44	48.4
≥ $768	14	15.4
Religiousness		
Religion		
Yes	85	93.4
No	6	6.6
What religion		
No religion	6	6.6
Catholic	44	48.4
Evangelical	35	38.5
Umbanda	3	3.3
Spiritist	3	3.3
Clinical		
COVID-19 infection		
Yes	7	7.7
No	84	92.3
Nutritional status		
Low weight	9	9.9

Eutrophic	34	37.4
Overweight	32	35.2
Obesity	16	17.6
Primary tumor location		
Genitourinary	9	9.9
Gastrointestinal	36	39.6
Hematologic	18	19.8
Lung	7	7.7
Head and neck	15	16.5
Central nervous system	1	1.1
Liver, pancreas, and biliary tract	3	3.3
Unknown primary site	2	2.2
Cancer staging		
I	2	2.2
II	11	12.1
III	37	40.7
IV	38	41.8
Not defined	3	3.3
Performance status		
PS-0	16	17.6
PS-1	52	57.1
PS-2	23	25.3
Type of treatment		
Curative	16	17.6
Adjuvant	11	12.1
Neoadjuvant	26	28.6
Palliative	38	41.8
Year of diagnosis		
2013	1	1.1
2017	2	2.2
2018	2	2.2
2019	2	2.2
2020	48	52.7
2021	36	39.6
Diagnosis during the pandemic		
No	7	7.7
Yes	84	92.3
Concurrent treatment		
No	70	76.9
Hormone therapy	4	4.4
Radiotherapy	17	18.7
Use of psychotropic drugs		
Yes	26	28.6

No	65	71.4
Visual Analogue Scale (VAS)		
Pain		
Yes	23	25.3
No	68	74.7
Pain intensity		
Mild (0–2)	72	79.1
Moderate (3–7)	18	19.8
Severe (8–10)	1	1.1
VAS score		
Mean (standard deviation)	1.11 (2.13)	
Minimum (maximum)	0 (9)	
Distress Thermometer (DT)		
Distress		
Yes (DT≥ 4)	45	49.5
No (DT<4)	46	50.5
DT score		
Mean (standard deviation)	3.81 (3.46)	
Minimum (maximum)	0 (10)	
Spiritual Well-Being Scale		
Religious well-being subscale score		
Mean (standard deviation)	57.87 (4.00)	
Minimum (maximum)	43 (60)	
Existential well-being subscale score		
Mean (standard deviation)	48.67 (7.03)	
Minimum (maximum)	30(60)	
Total score		
Mean (standard deviation)	106.54 (9.06)	
Minimum (maximum)	75.00 (120)	

Table S2. Distribution of problems according to the Distress Thermometer and Problem List for Patients classification, and significance of differences between patients with and without distress starting chemotherapy in the pandemic. Rio de Janeiro, Brazil, 2021, (*n* = 91)

**Variables**	**No distress**		**Distress**		**Total**		**p-value**
	**n**	**%**	**n**	**%**	**n**	**%**	
Practical problems							
Child care							
Yes	11	23.9	12	26.7	23	25.3	0.763(1)
No	35	76.1	33	73.3	68	74.7	
Housing							
Yes	16	34.8	16	35.6	32	35.2	0.930(1)
No	30	65.2	29	64.4	59	64.8	
Insurance/financial							

Yes	21	45.7	26	57.8	47	51.7	0.247(1)
No	25	54.4	19	42.2	44	48.4	
Transportation							
Yes	23	50	20	44.4	43	47.3	0.595(2)
No	23	50	25	55.6	48	52.8	
Work/school							
Yes	21	45.7	24	53.3	45	49.5	0.460(1)
No	25	54.4	21	46.7	46	50.6	
Family problems							
Children							
Yes	2	4.4	6	13.3	8	8.8	0.130(1)
No	44	95.7	39	86.7	83	91.2	
Partner							
Yes	1	2.2	8	17.8	9	9.9	0.032(2) *
No	45	97.8	37	82.2	82	90.1	
Emotional problem							
Depression							
Yes	6	13.0	17	37.8	23	25.3	0.006(3) *
No	40	87.0	28	62.2	68	74.7	
Fears							
Yes	12	26.1	23	51.1	35	38.5	0.014(3) *
No	34	73.9	22	48.9	56	61.5	
Nervousness							
Yes	22	47.8	29	64.4	51	56.0	0.167(2)
No	24	52.2	16	35.6	40	44.0	
Sadness							
Yes	15	32.6	29	64.4	44	48.4	0.002(3) *
No	31	67.4	16	35.6	47	51.7	
Worry							
Yes	28	60.9	40	88.9	68	74.7	0.002(1) *
No	18	39.1	5	11.1	23	25.3	
Loss of interest in usual activities							
Yes	21	46.7	14	31.1	35	38.5	0.154(1)
No	25	55.6	31	68.9	56	61.5	
Spiritual/religious concerns							
Yes	12	26.1	11	24.4	23	25.3	0.857(1)
No	34	73.9	34	75.6	68	74.7	
Physical problems							
Appearance							
Yes	11	23.9	11	24.4	22	24.2	0.953(1)
No	35	76.1	34	75.6	69	75.8	
Bathing/dressing							

Yes	3	6.5	4	8.9	7	7.7	0.714(3)
No	43	93.5	41	91.1	84	92.3	
Breathing							
Yes	13	28.3	14	31.1	27	29.7	0.766(1)
No	33	71.7	31	68.9	64	70.3	
Changes in urination							
Yes	12	26.1	12	26.7	24	26.4	0.004(1) *
No	34	73.9	33	73.3	67	73.6	
Constipation							
Yes	13	28.3	16	35.6	29	31.9	0.455(1)
No	33	71.7	29	64.4	62	68.1	
Diarrhea							
Yes	7	15.2	12	26.7	19	20.9	0.250(2)
No	39	84.8	33	73.3	72	79.1	
Eating							
Yes	18	39.1	9	20	27	29.7	0.046(1) *
No	28	60.9	36	80	64	70.3	
Fatigue							
Yes	15	32.6	15	33.3	30	33.0	0.941(1)
No	31	67.4	30	66.7	61	67.0	
Feeling swollen							
Yes	10	21.7	11	24.4	21	23.1	0.759(1)
No	36	78.3	34	75.6	70	76.9	
Fevers							
Yes	4	8.7	1	2.2	5	5.5	0.161(2)
No	42	91.3	44	97.8	86	94.5	
Getting around							
Yes	9	19.6	12	26.7	21	23.1	0.421(1)
No	37	80.4	33	73.3	70	76.9	
Indigestion							
Yes	5	10.9	11	24.4	16	17.6	0.089(1)
No	41	89.1	34	75.6	75	82.4	
Memory/concentration							
Yes	14	30.4	14	31.1	28	30.8	0.940(1)
No	32	69.6	31	68.9	63	69.2	
Mouth sores							
Yes	3	6.5	4	8.9	7	7.7	0.672(1)
No	43	93.5	41	91.1	84	92.3	
Nausea							
Yes	11	23.9	8	17.8	19	20.9	0.472(1)
No	35	76.1	37	82.2	72	79.1	
Nose dry/congested							

Yes	10	21.7	7	15.6	17	18.7	0.449(1)
No	36	78.3	38	84.4	74	81.3	
Pain							
Yes	21	45.7	20	44.4	41	45.1	0.908(1)
No	25	54.4	25	55.6	50	55.0	
Sexual							
Yes	10	21.7	13	28.9	23	25.3	0.433(1)
No	36	78.3	32	71.1	68	74.7	
Skin dry/itchy							
Yes	11	23.9	16	35.6	27	29.7	0.224(1)
No	35	76.1	29	64.4	64	70.3	
Sleep							
Yes	19	41.3	23	51.1	42	46.2	0.348(1)
No	27	58.7	22	48.9	49	53.9	
Tingling in hands/feet							
Yes	9	20.0	13	28.9	22	24.2	0.229(1)
No	37	82.2	32	71.1	69	75.8	

(1) Chi-square; (2) likelihood ratio; (3) Fisher's exact test; * $p \leq 0.05$ (statistically significant)
